# Personality theory: New factors to incorporate in public decision‐making in communities

**DOI:** 10.1002/hcs2.43

**Published:** 2023-04-19

**Authors:** Mengyao Yan, Jinzi Zhang, Pu Ge, Yibo Wu

**Affiliations:** ^1^ School of Health Policy and Management Chinese Academy of Medical Sciences & Peking Union Medical College Beijing China; ^2^ School of Humanities and Social Sciences Harbin Medical University Harbin China; ^3^ Institute of Chinese Medical Sciences University of Macau Macao China; ^4^ School of Public Health Peking University Beijing China

**Keywords:** decision‐making, personality, policy

## Abstract

**Background:**

This study explored the effects of personality factors on public behavioral decision‐making.

**Methods:**

We examined the literature on personality theory based on triadic interaction decision theory, and summarized and compared the findings with studies of the Big Five personality characteristics. A literature review method was used to explore the implications of personality theory for public decision‐making in Chinese communities.

**Results:**

Individuals with high neuroticism can be targeted by influential communicators. Individuals with high extraversion can influence decision‐making through interpersonal relationships. Individuals with high levels of openness can be influenced by the development of novel activities. Conscientious individuals respond to scientific and rational knowledge. Individuals with high agreeableness can be influenced by groups.

**Conclusions:**

Personality traits can influence behavioral decisions and can have positive or negative effects on behavioral outcomes. For people with different personality traits, social actors and social activity communicators should formulate targeted measures according to the classification of personality traits. The current findings have implications for enriching research perspectives and approaches to public community decision‐making.

## 1 INTRODUCTION

**Figure 1:**
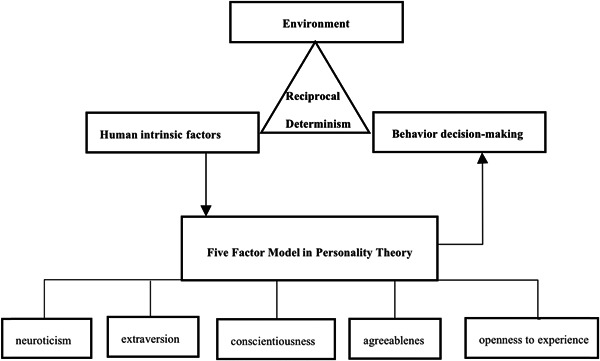
Personality and behavioral decision‐making framework.

Social phenomena occur as a result of social action, and social action begins with behavioral decision‐making. A previous study examined how informed choice policies and issues of choice and decision‐making are thematized around how policies and practices for prenatal screening services are developed through conversations between pregnant women and health professionals. This study provided a useful perspective regarding the relationship between choice and decision‐making [1]. In April 2020, China entered a phase of regular epidemic prevention and control. After a comprehensive assessment by national experts, China chose to implement a “dynamic zero” policy and mass vaccination, with a view to implementing herd immunity. In contrast, in the early stage of the epidemic, the United Kingdom chose “infection” as an approach to achieve herd immunity. The formulation of policies at the government level is a decision‐making challenge, and, in the face of vaccination, the public exhibits a range of responses, such as vaccine hesitation and vaccination preference, constituting a choice decision problem at the public level. According to theoretical research in behavioral economics, parents, or caregivers of children with compromised health were influenced in their behavioral decisions to seek medical care via choice preferences [2]. The problem of behavioral decision‐making has been studied in the field of behavioral research, and is the starting point of all actions, from individuals, to groups, national populations, and the global population, including small‐scale issues related to food, clothing, housing, and transportation, and large‐scale issues related to national and global development. Appropriate behavioral decisions will guide the results of individual decisions and the choice of national development paths in the right direction, whereas inappropriate behavioral decisions will hinder the development of individuals and nations. Over the past 20 years, several Nobel Prize winners have worked on behavioral decision‐making, helping governments and actors at all levels to formulate public policies and solve social problems [3]. Behavioral decisions are subject to multiple factors, including environmental, economic, and psychological factors. The current study focused on psychological factors. Behavioral decisions can produce three outcomes: confrontation, silence or nonengagement, and altruistic behavior focused on helping others. Personality has been a hot research topic in the fields of psychology, behavioral economics, and organizational behavior [4]. Personality has been described in psychological research as a set of stable psychological characteristics that is not easily changed, and is reported to be related to individuals’ psychological and behavioral responses to situations. Therefore, personality theory is more widely applied in psychology than in the field of management. The current study explored the mechanisms by which personality factors influence public behavioral decision‐making, suggesting that policy implementers should proactively incorporate personality factors in the process of policy implementation to influence public decision‐making (Figure 1).

## 2 PERSONALITY IS AN IMPORTANT DETERMINANT OF VARIOUS ATTITUDES AND BEHAVIORS

The relationships between personality, behavior, and environment can be explained by Bandura's theory of reciprocal determinism. The triadic interaction of determinism incorporates advanced theories, such as social construction and humanism, which differentiate Bandura's theory in the field of behaviorist psychology. Bandura describes interactions and mutual decision‐making between behavior, human intrinsic factors, and the environment, behavior, and the environment as interacting decision factors, and human intrinsic factors and behavior as interacting decision factors [5]. First, the interplay between behavior and environment can be explored. Behavior partially determines changes in the environment and is also influenced by the environment. Policy issues occur in particular sociopolitical–economic–cultural contexts. In a meeting agenda, policy options are then selected that are appropriate and compatible with the context. The general social context creates the general environment for policy decisions, political implementation of policy is practiced, and the behavior of the policy transforms the development of society, and thus to a certain extent also determines changes in the environment. Second, interactions occur between human internal factors and the environment. Human internal factors are involved in drawing selective influences from the environment. Additionally, humans are social animals, and the social context also affects human internal factors. Human internal factors are formed in a general social context, but they are not fully subject to the current social context. The social context changes, but the formed internal factors are more stable and do not change easily. Finally, the interaction between human internal factors and behavior can be explored. Human behavior is governed by people, and people are influenced by numerous factors when making behavioral decisions. In the long term, the results of human behavior in certain stages of a person's life will prompt changes in their internal state. Each individual is influenced by many internal factors. The current study focused on personality and human behavior.

## 3 THE FIVE‐FACTOR MODEL IN PERSONALITY THEORY

Although there are many schools of thought regarding personality theory, there is a relatively broad consensus regarding a model of personality factors. Cattell [6] proposed the initial Big Five model of personality, which is also known as the five‐factor model. In 1993, Goldberg's five‐factor model achieved widespread acceptance and has been applied internationally. The Big Five personality model is a relatively mature model and is generally accepted by the academic community. In the current study, we used the Big Five personality model as an example to explore the mechanisms by which personality influences behavioral decision‐making. The Big Five personality scale is based on the Big Five personality theory, and quantifies human psychology and personality to further understand the relationship between psychology and personality. The Big Five personality model is a consensus model, with five factors: neuroticism, extraversion, openness, conscientiousness, and agreeableness. John et al. [7] defined the five factors of the Big Five personality as follows. Neuroticism is characterized by a tendency to experience negative emotions. Typical adjectives describing neuroticism are temperamental, tense, and impressionable. Extraversion indicates the amount and intensity of sensation‐seeking and interpersonal relationships, with typical adjectives being sociable, confident, and energetic. Openness refers to autonomous thinking, a willingness to investigate unfamiliar ideas, and a tendency to try new things; typical adjectives describing openness are inquisitive, philosophical, and innovative. Accountability describes the quality of interpersonal interactions on a continuum from social antagonism to empathy, with typical adjectives being kindness, thoughtfulness, and generosity. Self‐awareness is characterized by responsibility, persistence, and self‐awareness, with typical adjectives describing self‐awareness being organized, responsible, and efficient. For example, according to the State Council's interagency taskforce responsible for the COVID‐19 epidemic, the vaccination rate of the entire Chinese population against the COVID‐19 vaccine exceeded 90% by the end of 2022. However, this still falls short of the rate required to achieve herd immunity. A study of the Big Five personality traits and vaccination predicted that people with neurotic personality traits would be more concerned about the COVID‐19 outbreak for a longer period of time and hold a negative attitude toward uncertain events. People with extroverted personality traits are confident and expressive, and tend to go against guiding principles. People with conscientious personality traits were expected to exhibit a higher level of compliance with preventive measures and a higher level of organizational discipline [8]. Personality characteristics, in the context of vaccine hesitation, are considered to influence the behavioral decisions of the public, and their subdivision is conducive to further refinement of policies and precise control.

## 4 PERSONALITY TRAITS INFLUENCE BEHAVIORAL DECISION‐MAKING

A previous study reported that members of the public with different personality traits exhibited different preferences regarding COVID‐19 vaccination. Open and extroverted respondents in the Big Five personality group exhibited the greatest willingness to vaccinate, and stable and self‐aware respondents considered the cost of vaccines to be the most important factor. In addition to studies in vaccine‐related fields, personality traits also play a role in agriculture, finance, and other fields. Several studies on personality traits and farmers’ entrepreneurial choices reported that cisgender traits inhibited farmers’ entrepreneurial motivation, and extraversion and openness significantly promoted farmers’ entrepreneurial participation [9]. Personality traits have been reported to influence whether older adults take COVID‐19 precautions. Studies have reported that older people with higher levels of openness, conscientiousness, and neuroticism are more willing to take protective precautions and less likely to contract COVID‐19. Some evidence also suggests that older adults with low levels of openness, conscientiousness, and neuroticism could benefit from prevention strategies tailored to these personality traits [10]. Furthermore, a study using the Big Five personality traits reported that presenteeism was higher among individuals with high levels of neurotic personality traits, with neurotic personality traits contributing to presenteeism to some extent. Therefore, nursing managers may be able to implement targeted measures to intervene to reduce presenteeism [11]. Overall, different personality traits can have a facilitating or inhibiting effect on behavioral decision‐making, and the same personality traits can vary in their effect on decision‐making choices across behaviors. Therefore, the influence of personality factors on behavioral decision‐making should be analyzed according to specific behavioral situations.

## 5 DISCUSSION

Communities must address the broadest group of people possible, and are often the policy implementers when it comes to making decisions for the general public. How can policy implementers use the influence of personality models among policy recipients? National governments formulate policies as a top‐level design, and local governments are guided by the top‐level design to formulate policies in their regions according to local conditions. Government policies apply to the general public, and the implementation of policies requires effective measures by social actors. Personality traits are core stable traits of individuals, and different personality traits affect public decision‐making. Therefore, policy implementers should classify policies according to personality traits, refine policies, and implement targeted measures for social activities, mass communication, and precise implementation. The current study focused on five personality factors in categories, as follows (Table 1):

1. Neuroticism: The results shown in the table indicate that this personality trait can be targeted by more influential communicators with personal emotional experiences and “storytelling” narratives, by adding topic tags with specific personality traits and pushing them to individuals through big data analysis.
2. Extraversion: For example, in health education for diabetic patients, peer support such as peer phone interventions and group interventions can be conducted for individuals with extroverted personalities, resulting in higher levels of adherence. In addition, the “Little Hands, Big Hands” series connects children and adults in a family as a unit, and indirectly promotes behavior change in adults through the education of children.
3. Openness: For example, in one region, when conducting the third vaccination campaign for the new crown pneumonia vaccine, a popular character called Bing Dwen Dwen was used in publicity material, which could further increase the vaccination rate among people with high levels of openness in their personality traits.
4. Conscientiousness: When social actors intervene, they can invite experts in the field to conduct scientific activities, and the government can publicize and report more scientific knowledge about the safety of vaccines to further improve the public's behavioral decisions.
5. Agreeableness: Community‐based interventions can be conducted in groups of adolescents, middle‐aged, and older people, with horizontal and vertical actions in various age groups, and using groups such as organizational teams.

**Table 1:** Characteristics of the five personality categories and related research.

Personality	Trait	Authors	Publishing year	Main findings
Neuroticism personality	The main thing is the degree of emotional stability and a tendency to negative emotions	Batson GJ, Mattew TR, Brannect BH	1995	Through altruistic tendencies, the connection with others for emotional experiences as well as well‐being empathy can detoxify the negative emotional experiences of neurotic personality^12^
		Wu F	2019	When conducting social movements for such personalities, social actors should start with empathy. The ability to empathize is a conscious transposition from objective reality, understanding the behavioral experience of the other person, capturing emotional characteristics, and reacting to unique individuals as well as altruistic actions^13^
		Duan P	2022	With the prevalence of online communication, online news dissemination is certainly one of the options for conducting communication. Studies have shown that platforms such as Xiaohongshu, Jitterbug, and Beeping are community‐oriented and emotional^14^
Extraversion personality	Good interpersonal relationships, self‐confidence, and good at expressing themselves.	Dennis CL	2003	Getting started with interpersonal relationships is an option for this type of personality, and peer education can be one of the breakthroughs in interpersonal relationships. Peer education refers to peer support from experienced people in a group of people facing the same problem or stress. Some studies have shown that peer education has been effective in the field of health care^15^
Openness	Curious and inclined to try new things	Min H‐W, Wu Y‐B, Sun X‐Y	2022	Some studies have shown that individuals with a high open personality are highly receptive to behavioral interventions for tobacco control.^16^ This further predicts the behavioral characteristics of an open personality.
Conscientiousness	Fair, organized, and cautious	Rostami M, Ahmadboukani S, Saleh Manijeh H.	2022	For such personalities, think twice before making behavioral decisions and need to be persuaded with scientific and reasonable knowledge, facts, and evidence.^17^
Agreeableness	Gentle, empathetic, and focused on organizational discipline and efficiency	Li Z‐M, Gao M, Chen X‐Y	2020	When social actors carry out social movements, they should focus on training the leaders of the organizations and communities, and then have the leaders deliver effective messages down the line. It has been found that differences in conscientious personality are influenced by age factors.^18^

## 6 CONCLUSION

In summary, personality traits can influence behavioral decisions, and can have a positive or negative impact on behavioral outcomes. Social actors or social communicators should develop targeted measures according to the classification of personality traits, and these interventions should be tailored to the local and contemporary context, so that government departments can further promote public decision‐making, reduce negative impacts, change behavioral attitudes, and promote positive decision‐making.

## AUTHOR CONTRIBUTIONS

**Mengyao Yan**: Project administration (lead); writing—original draft (lead); writing—review and editing (lead). **Jinzi Zhang**: Writing—original draft (supporting). **Pu Ge**: Writing—original draft (supporting). **Yibo Wu**: Conceptualization (lead); funding acquisition (equal); supervision (lead).

## CONFLICT OF INTEREST STATEMENT

The authors declare no conflict of interest.

## ETHICS STATEMENT

The authors have nothing to report.

## INFORMED CONSENT

The authors have nothing to report.

## Data Availability

Data sharing not applicable to this article as no datasets were generated or analyzed during the current study
